# Patients’ support for health information exchange: a literature review and classification of key factors

**DOI:** 10.1186/s12911-017-0436-2

**Published:** 2017-04-04

**Authors:** Pouyan Esmaeilzadeh, Murali Sambasivan

**Affiliations:** 1 Department of Information Systems and Business Analytics, College of Business, Florida International University, Miami, Florida USA; 2 Taylor’s Business School, Taylor’s University Lakeside Campus, Subang Jaya, Malaysia

**Keywords:** *Health information exchange*, *Perceived benefits*, *Perceived concerns*, *Patient participation*, *Patient characteristics*, *Consent preference*

## Abstract

**Background:**

Literature indicates that one of the most important factors affecting the widespread adoption of Health Information Exchange (HIE) is patient support and endorsement. In order to reap all the expected benefits of HIE, patients’ acceptance of technology is a challenge that is not fully studied. There are a few studies which have focused on requirements of electronic medical information exchange from consumers’ views and expectations. This study is aimed at reviewing the literature to articulate factors that affect patients to support HIE efforts.

**Methods:**

A literature review of current studies addressing patients’ views on HIE from 2005 was undertaken. Five electronic research databases (Science Direct, PubMed, Web of Science, CINAHL, and Academic Search Premiere) were searched to retrieve articles reporting pros and cons of HIE from patients’ opinion.

**Results:**

One hundred and ninety six articles were initially retrieved from the databases. Out of 196, 36 studies met the inclusion criteria and were fully reviewed. Our findings indicate that patient’s attitude toward HIE is affected by seven main factors: perceived benefits, perceived concerns, patient characteristics, patient participation level in HIE, type of health information, identity of recipients, and patient preferences regarding consent and features.

**Conclusions:**

The findings provide useful theoretical implications for research by developing a classification of significant factors and a framework based on the lessons learned from the literature to help guide HIE efforts. Our results also have fundamental practical implications for policy makers, current and potential organizers of HIEs by highlighting the role of patients in the widespread implementation of HIE. The study indicates that new approaches should be applied to completely underline HIE benefits for patients and also address their concerns.

**Electronic supplementary material:**

The online version of this article (doi:10.1186/s12911-017-0436-2) contains supplementary material, which is available to authorized users.

## Background

The Health Information Exchange (HIE) is an important component of the Health Information Technology (HIT) infrastructure that is designed to facilitate electronic movement of patients’ health information among healthcare organizations during the care process [1]. HIE promises several potential benefits through improved quality, safety and efficacy of healthcare services [2]. A number of governments around the world (such as U.S., Australia and New Zealand) support the exchange of patient information between various stakeholders [3]. The federal “meaningful use” program in U.S. is intended to provide incentives for the adoption and implementation of Electronic Health Records (EHRs) among healthcare providers to promote the online exchange of patients’ information [4]. Huge investments are being made by the federal government to encourage the use of interoperable HIT to enable HIE among healthcare providers [5]. The Health Information Technology for Economic and Clinical Health (HITECH) Act was passed in 2009 in U.S. to financially support the nationwide adoption of HIT and HIE [6]. The widespread use of HIE plays an important role in the healthcare reform [7]. Exchange of patient health information was expected to increase exponentially in the current decade [8]. However, there are still many challenges regarding the use of HIE in UK, U.S., Australia and Sweden [9]. The most cited impediments are technical limitations, financial constraints, lack of interoperability, privacy, and security concerns [2]. Furthermore, there are a number of stakeholders in the healthcare sector who can be producers and users of health information. Obtaining coordination among all the stakeholders can be very challenging due to legal, safety, security, and operational issues [10]. One of the most critical stakeholders is healthcare consumers because their consent is required for sharing their health information. The patients’ attitude towards sharing their personal health information can affect the design of future health information systems [3]. Therefore, studying their views on the system and addressing potential disparities in HIE implementation is very essential [11].

Patel et al. [12] indicate that consumers’ support of HIE is influenced by two main variables: (1) potential benefits of HIE and (2) privacy and security concerns. Recent studies state that patients are very positive about the use of HIE systems by healthcare providers due to potential gains but they are also cautious about possible privacy and security breaches [13]. HIE involves technologies such as EHRs which support the capture and sharing of electronic information for healthcare purposes [14]. On the other hand, although, EHR systems may increase the accessibility of patients’ records, studies show that the potential threats to the confidentiality of patients’ medical information are more controversial [15]. Patient information breaches have created a serious challenge and harmed patients due to unauthorized disclosure of their information [16]. As a result, Sankar et al. [17] report that a group of patients may not seek or continue treatment if the confidentiality of their medical records is not satisfactory. The increasing levels of concern about personal privacy have turned the attention of research into consent issues and the development of electronic systems to better control access to patient information [18].

In order to reap the potential benefits of HIE, patients support to allow exchange of their personal health information will be crucial [19]. Widdett et al. [3] have indicated that very little research has been conducted about attitudes of patients towards HIE. Moreover, little work has addressed privacy issues associated with HIE from patients’ perception. Most of the articles on privacy issues were conducted from clinicians’ perspectives or discussed legal or regulatory issues. The other gap in the literature is lack of research on the attitude of ordinary patients since the majority of articles concentrated on a specific group of patients with sensitive health-related information such as patients with HIV. According to Park et al. [10], there is a gap in the literature about the type and nature of information that patients prefer to be shared using HIE efforts. It is also unclear what expected benefits will lead patients to endorse the HIE technology and what perceived concerns will make them reluctant about online electronic exchange of their health information by healthcare providers. Due to the importance of security issues, privacy concerns, confidentiality of personal health information, and providing consent for electronic transmission of clinical information, policymakers have begun to focus on HIE from patients’ views. However, little is known about patients’ attitudes toward exchange process, consent systems, and their participation and role in HIE [9].

To fill these gaps in the literature, this article is aimed at reviewing the current literature to better articulate the attitude of patients towards HIE. This research attempts to elaborate how well patients understand the possible benefits and adverse effects of data exchange among healthcare providers. Consequently, the main research question posed by this study is: what factors do encourage patients to make their decision to support HIE? This research also attempts to answer what barriers will impede them from endorsing the electronic exchange of their health information. In line with these research questions, we propose a classification of key factors that affect patients’ willingness to support HIE.

## Methods

### Eligibility criteria

We reviewed current theoretical and empirical studies related to HIE from consumers’ views in various healthcare settings. All retrieved studies which published in the refereed journals from the year 2005 and in English language were included in the review. We limited our search to the last 11 years since we observed that many studies from 2005 onwards used the concept of HIE and sufficiently discussed issues related to patients expectations and their attitude toward HIE. Studies that were editorials, commentaries, opinion papers or articles without an abstract were excluded from further consideration.

### Search strategy

The main objective of this study was to undertake a literature review of existing studies on patients’ perception of HIE. To identify the right set of key words and databases, the authors used the help of a health librarian. To meet the objective, the studies were mainly searched in five electronic research databases of Science Direct, PubMed, Web of Science, CINAHL, and Academic Search Premiere. The main keywords used for searching articles were “Health Information Exchange”, “HIE”, “Patients”, “Consumers”, “Participation”, “Involvement”, “Role”, “Attitude”, “Experience”, “View”, “Concerns”, and “Benefits” (see Additional file 1 for details of full strategy). We continued searching until no new studies were found in light of the selection criteria.

### Quality assessment

We reviewed both qualitative and quantitative peer-reviewed original research studies. Quality appraisal of articles is common in literature reviews to measure the quality or veracity of each study included in the review. To address this, we used a quality assessment tool for integrative reviews. The tool assesses quality and study rigor based on four factors: study type, sampling method, data collection method detail, and analysis [20, 21]. The possible score generated by this tool ranges between 4 (qualitative design, sampling, and data collection not explained, and narrative analysis) to 13 (quantitative experimental design, random sampling, data collection explained, and inferential statistics) [20] (see Additional file 2 for details of quality scoring of included studies). To ensure the strength of evidence and quality of the studies reported, authors independently scored the articles in rounds and disagreements were resolved by discussion until more than 95% agreement was achieved.

### Selection of studies

**Fig. 1:**
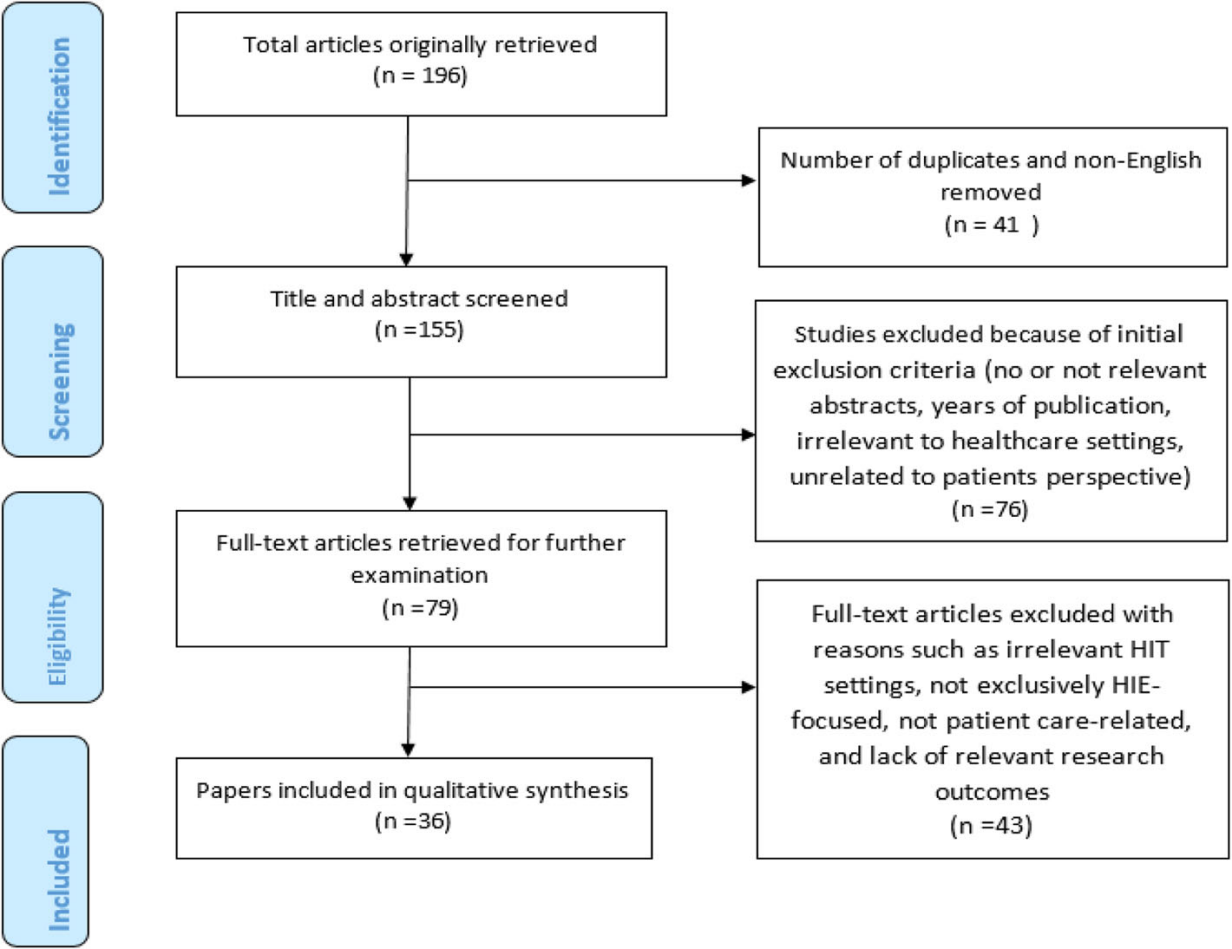
Literature search flow diagram

Through database searching, 196 articles published during and after 2005 were retrieved. There were 41 duplicates and non-English articles that were removed resulting in 155 articles. The titles and abstracts of these 155 papers were screened and 76 papers were excluded based on the initial exclusion criteria (no or not relevant abstracts and not relevant HIT settings). To be clear, we included articles related to systems such as EHRs, Personal Health Records (PHRs) and HIE architectures and excluded articles that just focused on settings such as Clinical Decision Support System (CDSS), Computerized Physician Order Entry (CPOE) and etc. The selected papers (79 studies) were reviewed in full and assessed for eligibility. To obtain the final set of papers, 43 papers were further excluded with reasons such as not exclusively HIE-focused, not patient care-related, lack of relevant research outcomes, or too general discussions with no clear theoretical and practical contributions. Finally, 36 papers were used in a qualitative synthesis and a summary of included papers are indicated in Table 2. Figure 1 depicts the study selection flow process.

## Results

### Characteristics of studies

Table 1 shows that the study design and type of methods used in the included studies. Fourteen studies applied a qualitative method such as focus group, direct observation or interview. Twenty one papers (58%) described their results using a quantitative study design. Only one study used a mixed method by including both survey and interview approach. This finding shows that the majority of included studies have supported their results using a survey to explore patients’ views about HIE.

**Table 1:** Study designs and methods of included studies

Study design	Method	Number of articles
Qualitative	Conceptual paper	7
	Literature review	2
	Focus group	2
	Direct observation, and interview	2
	Direct observation, interview, and focus group	1
Quantitative	Survey	21
Mixed	Survey and interview	1
	Total	36

**Fig. 2:**
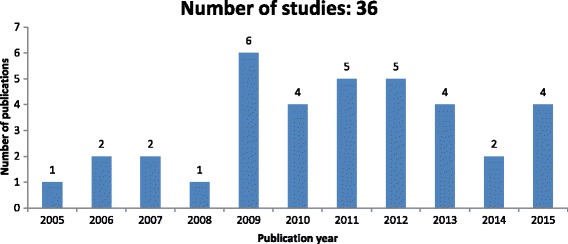
Distribution of publications per year across the period studied

This study conducted an analysis of the distribution of included publications per year across the period studied in order to show the trend of research over the years. Figure 2 depicts the year wise distribution of all 36 articles from 2005 (January) to 2015 (December). The graphical representation of Fig. 2 indicates the increasing number of research articles published over the last eleven years. The year 2009 has the highest number (6) of published articles and years 2011 and 2012 have the second highest (5). In 2014, there are only two articles selected based on the inclusion criteria but it does not contradict that the publication of journal articles has evolved over time. This increasing trends highlights the increasing attention directed toward the important role of public attitudes and preferences in nationwide implementation of HIE.

**Fig. 3:**
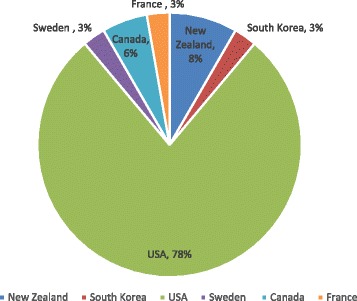
Geographic location of publications

Figure 3 presents the information on the geographical location of publications on HIE from patients’ perception. An original contribution of this section is to show that USA is the country leader where a successful implementation of HIE depends on users awareness and support. In fact, this finding can be explained by the current effort made by the HIE policy makers in USA to increase public awareness of HIE. The policy makers in New Zealand and Canada are also concerned about the role of public support in the success of HIE projects. We observed a lack of interest in many underdeveloped and developing countries.

### Main findings

In order to reflect a synthesis of the 36 selected studies and explain how these papers lead to reported results and proposed classification, the extracted information from review of included articles are presented in this section. Table 2 provides an overview of the authors, publication year, method, targeted population, sample size, and main results of all the 36 articles.

**Table 2:** Review of included studies

#	Reference	Country	Method	Targeted population, sample size	Main results and recommendations
1	Agaku et al. (2014) [27]	USA	Quantitative study (survey)	US adults aged ≥18 years, 3959 respondents	Majority of respondents expressed data breach concerns when their protected health information was being exchanged between healthcare professionals by fax or electronically. Respondents were more likely to withhold their medical information when they perceived they knew very little about how their medical records were used.
2	Ancker et al. (2012) [7]	USA	Quantitative study (random-digit-dial telephone survey)	Adult New York State residents, 800 respondents	Majority of respondents supported HIE among healthcare providers owing to improving quality of care. They also endorsed physicians to access their data without requesting for consent in emergencies. Privacy and security concerns were also raised by 68% of respondents. The study stated that consumers were supportive of HIE whether the architecture was centralized, federated, point-to-point or hybrid model.
3	Ancker et al. (2013) [23]	USA	Quantitative study (survey)	Adults (national wide), 100 respondents	Majority of respondents believed that HIE could improve healthcare quality. Respondents whose physicians used EHR were more likely to support HIE.
4	Beard et al. (2012) [31]	N/A	Literature review	Patients	The main challenges associated with HIE: cost and security concerns, confusion around responsibilities and rights of the various players, liability issues and tensions between flexible access to data and flexible access to physicians.
5	Caine & Hanania (2013) [32]	USA	Qualitative and quantitative (survey, interview)	Patients, 30 respondents	Two factors affecting patients to support sharing of their medical records were type of information and type of recipient. Patients desired more privacy control over which health information should be shared with whom. Patients also preferred less sharing of sensitive versus less-sensitive information.
6	Campos-Castillo & Anthony (2015) [40]	USA	Quantitative study (survey)	US adults (national wide), 4753 respondents	Patients with a provider using an EHR was more likely to withhold personal information because of privacy concerns. US immigrants were more prone to ever withhold information from a provider.
7	Chhanabhai & Holt (2007) [29]	New Zealand	Quantitative (cross-sectional survey)	Health consumers visiting healthcare practices, 300 respondents	Participants were highly worried about the security and privacy of their online health records. Participants were concerned about hackers (79.4%), vendor access (72.7%), and malicious software (68%).
8	Dhopeshwarkar et al. (2012) [70]	USA	Quantitative study (random-digit-dial telephone survey)	Residents in the Hudson Valley of New York State, 170 respondents	Majority of consumers desired to be asked for a permission before various parties, including their clinician, could see their health records through HIE. They also asked to check who has viewed their information (86%), to stop electronic storage of their data (84%), to stop all viewing (83%), and to select which parts of their health records can be shared (78%).
9	Dimitropoulos & Rizk (2009) [60]	USA	Conceptual	Policies, and state laws related to the privacy and security of health information	The study proposed a number of policies to protect health information and facilitate HIE such as: consent and permission, standard authentication and audit policies, harmonizing state privacy laws, and consumer education and engagement.
10	Dimitropoulos et al. (2011) [6]	USA	Quantitative study (random-digit-dial telephone survey)	English-speaking adults, 1847	Majority of respondents expressed that they were concerned about privacy (70%) and security of HIE (75%). Concerns were meaningfully greater among employed individuals, 40 to 64 years old, and minorities. Around 60% just supported HIE for treatment purposes. 52% desired to choose which providers should access and share their data.
11	Furukawa et al. (2014) [53]	USA	Quantitative study (mail survey)	Office-based physicians, nationwide	HIE with other providers was mainly limited in office settings, with only 14% sharing data with providers outside their organization. More policies are required to support HIE and patient engagement in data exchange.
12	Galpottage & Norris (2005) [61]	New Zealand	Conceptual	Patients	This study described the four characteristics of e-consent systems as protecting privacy, informing patients, capturing permission, and releasing information. This study recommended that consumers should be able to see who has accessed what part of their health information and why.
13	Grande et al. (2013) [59]	USA	Quantitative study (online survey)	Adults, 3336	Willingness to share electronic health information is affected by purpose for using information (research, quality improvement, or commercial marketing) and users (university hospitals, commercial enterprises, or public health departments). Data sensitivity was not a significant factor.
14	Hincapie et al. (2011) [46]	USA	Qualitative study (focus group)	Physicians, 29	Respondents reported that detecting drug-seeking behavior and doctor shopping, preventing duplicative testing, and increased efficiency of clinical information gathering were the most important benefits of HIE. The limited availability of data in the HIE system was mentioned as the most important disadvantage.
15	Kaelber & Bates (2007) [66]	USA	Conceptual	Patients	HIE can improve patient safety through improved medication information processing, improved laboratory information processing, improved radiology information processing, improved communication among providers, improved communication between patients and providers, and improved public health information processing
16	Kim et al. (2015) [14]	USA	Quantitative study (random-digit-dial telephone survey)	Californians, 800	Healthcare consumers believed that HIE would aggravated privacy and security concerns. They desired more transparency in HIE such as individual control, who can access, and the purpose for use of data. Respondents were more prone to share deidentified health information for research purposes.
17	Kullberg et al. (2015) [50]	Sweden	Quantitative analysis (survey)	Patients with cancer, 104	Majority of patients in oncological inpatient care were not satisfied with information exchange and information provision coming from doctors and nurses. This deficits increased patient safety risks such as medication errors and falls. Better policies for information exchange are required.
18	Letrilliart et al. (2009) [43]	France	Quantitative analysis (survey)	Patients, oncologists, general practitioners, nurses and other professionals, 48 members	Shared medical records for breast cancer patients would be organized in a way that patients, physicians, medical auxiliaries and other healthcare professionals were authorized to insert a piece of information. Lack of interactions, the complexity of the record, and threats to the confidentiality of patient sensitive data were the main concerns related to shared records.
19	McGraw et al. (2009) [55]	USA	Conceptual	HIE systems	This study recommended that to build public trust into HIE, a comprehensive privacy and security framework is needed to set clear rules for access to, use of, and disclosure of personal health information for all entities engaged in HIE. This framework also requires adequate oversight and accountability.
20	O’Donnell et al. (2011) [11]	USA	Quantitative analysis (telephone survey)	English-speaking residents of the Hudson Valley of New York, 170	Individuals who were caregivers for chronically ill patients were more likely to support physicians who used HIE. Individuals who earn more than $100,000 yearly were also more prone to support physician HIE. This study showed that males who used the internet frequently were more likely to endorse HIE.
21	Or & Karsh (2009) [38]	N/A	Systematic literature review	Patients	This study showed that majority of existing literature focused on patient-related factors to predict acceptance of consumer health information technology. The patient factors including sociodemographic characteristics, health- related variables, and prior experience with computer/health technology were the most cited factors.
22	Park et al. (2013) [10]	South Korea	Quantitative (longitudinal survey)	Patients, 730 (first round), 306 (second round)	Majority of respondents were willing to accept HIE due to improved quality and reduced healthcare bills, in spite of information safety and security concerns. People who experienced the HIE were more comfortable with the process of obtaining consent. This study showed that males respondents in their 40s and 50s were more likely to endorse HIE.
23	Patel et al. (2011) [68]	USA	Quantitative analysis (survey)	low-income, ethnically diverse consumers, 214	This study showed that a higher proportion of white and non-Hispanics (69%) expressed support for HIE compared to non-White or Hispanic individuals. Around 61% expressed support for HIE amongst their providers. This study recommended that considering cultural and socio-economic issues can be vital for achieving widespread support for HIE.
24	Patel et al. (2012) [12]	USA	Quantitative analysis (survey)	English speaking adults, 117 respondents	The more consumers felt potential benefits of HIE, they became more willing to support HIE. College education and prior experience using the internet could affect the level of consumers’ support for HIE. This study recommended that better policies should be made to demonstrate potential benefits of HIE and address privacy and security issues especially for individuals who are less educated.
25	Shield et al. (2010) [45]	USA	Qualitative study (observation, interview, focus group)	Patients, 170 clinical encounters, three focus groups with clinic nurses	Implementation of EHR (as the most important requirement of HIE) improved the level of patients’ trust in their relationship with physicians. It also fostered the sharing of medical information. Patients also reported the concerns for hacking, lost records, confidentiality breaches, technological malfunction, and viruses.
26	Simon et al. (2009) [9]	USA	Qualitative study (focus group discussions)	Adult community members, 64	The three main issues that emerged from the focus group discussions were concerns about privacy, security and misuse of health data, the potential benefit of HIE to a person’s health and safety, the desire for more information, and education about the consent process. This study recommended that clear educational materials are required to engage consumers in HIE.
27	Tang et al. (2006) [58]	USA	Conceptual	N/A	Health information should be shared with patients in ways that enables them to understand and to act on the information contained in their records. Although this helps individuals access their health information, the implementation and use of this system is challenging because individuals have various level of health literacy.
28	Teixeira et al. (2011) [42]	USA	Quantitative analysis (survey)	Persons living with HIV/AIDS, 93	Majority of respondents were more willing to share their personal health information with clinicians involved in their care and less likely to share with non-clinical staff.
29	Tripathi et al. (2009) [19]	USA	Conceptual	Patients	Privacy concerns and consent issues were identified as the most important design criteria for the HIE initiatives. This study recommended that there should be a balance between privacy protection procedures and availability as well as accuracy of patients’ medical information to improve the quality, safety, and efficiency of care.
30	Unertl et al. (2012) [22]	USA	Qualitative study (observation and interview)	Six emergency departments and eight ambulatory clinics, 180 h	Patient-provider trust was considered as an important factor in HIE systems. The reliability and accuracy of patient medical history reports shared through HIE was a big concern for users. This study recommended that improving HIE adoption depends on understanding the needs of different users.
31	Vest & Gamm (2010) [1]	USA	Conceptual	HIE stakeholders	Collecting patients’ health information into a single repository caused security and privacy concerns from patients and control and ultimate usage concerns from providers. HIE were mostly supported by chronically ill patients. The value of HIE should be measured in terms of benefits to all participants (patients, providers, payers, and communities).
32	Wang et al. (2015) [24]	USA	Quantitative analysis (survey)	physicians, medical record staff, and patients, 379 respondents	Patients needed more education and communication about the systems that store and exchange their medical information. This study reported that patients believe their privacy may be violated and their privacy should be protected through consent procedures. They expect that the system should improve their relationship with physicians.
33	Wen et al. (2010) [28]	USA	Quantitative analysis (survey)	US adults	Hispanic individuals who aged below 65 and used internet frequently were more likely to use the internet to keep track of their medical information through their PHRs. Males who aged above 45 years old were more willing to support HIE. This study recommended that consumer concerns regarding the security of HIE should be addressed.
34	Whiddett et al. (2006) [3]	New Zealand	Quantitative analysis (survey)	Adult primary-care patients, 200	Identity of recipients, level of anonymity, and type of information impacted patients’ attitude towards sharing their health information. They were more likely to share their information between healthcare professionals. Patients were less likely to share their personal information.
35	Wiljer et al. (2008) [47]	Canada	Qualitative study (workshop)	HIT experts, 45 participants	This study found that providing a clear definitions for privacy, security and confidentiality can help the implementation of EHRs. The study recommended that patient education, engagement and empowerment can also help consumers better understand the purpose of EHRs.
36	Wright et al. (2010) [25]	USA	Quantitative (a cross-sectional mail survey)	licensed physicians, 1043	Majority of physicians believed that HIE would improve quality of patient care, reduce healthcare costs and save time but they were also concerned about privacy of patients’ data.

### Classification of results

A qualitative method that usually used for coding descriptive transcripts was applied in this article to classify the results. The authors used a descriptive and narrative synthesis of the findings to understand the different types of factors affecting consumers to support (reject) HIE. The similarities and differences of the results were compared and contrasted in greater depth to identify the key factors. Then, the authors generated two factor lists individually which had 85% agreement rate. To create a more reliable classification and capture the influential factors from the included studies, a consent was achieved by the authors to report the factors which were mentioned by at least 20% of the reported studies. As a result, seven themes were extracted and all of them met the requirement because they were indicated by at least 20% of the studies (Table 3). Afterwards, authors selected consistent names for the agreed-upon factors. The seven factors are: perceived benefits of HIE, perceived concerns regarding HIE, patient characteristics, patient participation level in an exchange process, type of health information, identity of recipients, and patient preferences regarding consent and features. The classification that is developed based on the literature review can highlight the key focus of the reported papers used for conducting this study.

**Table 3:** Review of seven factors in included studies

#	Reference	Main factors retrieved from the literature						
		Perceived benefits	Perceived concerns	Patient characteristics	Level of patient participation in HIE	Types of health information to be exchanged	Types of recipients	Patients preferences regarding consent
1	Agaku et al. (2014) [27]	✖	✓	✖	✖	✖	✖	✓
2	Ancker et al. (2012) [7]	✖	✖	✓	✓	✓	✖	✓
3	Ancker et al. (2013) [23]	✓	✓	✓	✖	✖	✖	✖
4	Beard et al. (2012) [31]	✖	✓	✖	✓	✖	✖	✖
5	Caine & Hanania (2013) [32]	✖	✓	✓	✖	✓	✓	✖
6	Campos-Castillo & Anthony (2015) [40]	✓	✓	✖	✓	✖	✖	✖
7	Chhanabhai & Holt (2007) [29]	✖	✓	✓	✖	✖	✖	✖
8	Dhopeshwarkar et al. (2012) [70]	✖	✓	✖	✓	✓	✓	✓
9	Dimitropoulos & Rizk (2009) [60]	✖	✓	✖	✖	✖	✖	✓
10	Dimitropoulos et al. (2011) [6]	✓	✓	✓	✓	✓	✓	✖
11	Furukawa et al. (2014) [53]	✖	✖	✖	✓	✖	✓	✖
12	Galpottage & Norris (2005) [61]	✓	✓	✖	✖	✖	✖	✓
13	Grande et al. (2013) [59]	✓	✓	✓	✖	✓	✖	✓
14	Hincapie et al. (2011) [46]	✓	✖	✖	✓	✓	✖	✖
15	Kaelber & Bates (2007) [66]	✓	✖	✖	✖	✖	✖	✖
16	Kim et al. (2015) [14]	✖	✓	✖	✓	✓	✖	✓
17	Kullberg et al. (2015) [50]	✖	✖	✖	✓	✖	✖	✖
18	Letrilliart et al. (2009) [43]	✓	✖	✖	✓	✖	✖	✖
19	McGraw et al. (2009) [55]	✓	✓	✓	✓	✓	✖	✓
20	O’Donnell et al. (2011) [11]	✓	✓	✓	✓	✖	✖	✖
21	Or & Karsh (2009) [38]	✖	✖	✓	✖	✖	✖	✖
22	Park et al. (2013) [10]	✓	✓	✓	✖	✖	✖	✓
23	Patel et al. (2011) [68]	✓	✓	✓	✓	✖	✖	✖
24	Patel et al. (2012) [12]	✓	✓	✓	✖	✖	✓	✓
25	Shield et al. (2010) [45]	✓	✓	✖	✓	✖	✖	✖
26	Simon et al. (2009) [9]	✓	✓	✓	✖	✖	✓	✓
27	Tang et al. (2006) [58]	✓	✓	✖	✓	✖	✖	✖
28	Teixeira et al. (2011) [42]	✖	✖	✓	✖	✓	✓	✖
29	Tripathi et al. (2009) [19]	✖	✓	✖	✓	✖	✖	✓
30	Unertl et al. (2012) [22]	✖	✖	✖	✓	✖	✖	✖
31	Vest & Gamm (2010) [1]	✓	✓	✖	✖	✖	✖	✖
32	Wang et al. (2015) [24]	✓	✓	✖	✓	✖	✖	✓
33	Wen et al. (2010) [28]	✓	✓	✓	✖	✖	✖	✖
34	Whiddett et al. (2006) [3]	✖	✓	✖	✖	✓	✓	✓
35	Wiljer et al. (2008) [47]	✓	✓	✖	✓	✖	✖	✖
36	Wright et al. (2010) [25]	✓	✖	✖	✖	✖	✖	✖
Number of Articles		21	26	15	19	10	8	14
Percentage of articles addressing the factor		58%	72%	42%	53%	28%	22%	39%

To show how these studies result in our classification, the extracted factors from the review of included articles are presented in this section. Table 3 provides an overview of the seven factors (the tick mark indicates that the authors in their paper investigated the desired factors and the cross mark indicates that the authors did not refer to the desired factors). The key findings of our analysis are: (1) 72% of the included articles address perceived concerns and risks; (2) 58% of the articles address perceived benefits and potential advantages of HIE; (3) 53% of the articles study level of participation of patients in the process of data exchange and interaction with care providers; (4) 42% of the articles explain patient characteristics; (5) 39% of the articles deal with patients’ preferences regarding consent; (6) 28% of the articles mention the types of information to be exchanged through HIE, and (7) 22% of the articles talk about types of recipients of health information through HIE.

## Discussion

A total of 36 articles met the inclusion criteria. In this section, we highlight the synthesis of included articles and discuss the results of the classification to outline patients support for HIE. This section also delineate the challenges, barriers, and facilitators related to the seven factors identified through the classification.

### Perceived benefits

Perceived benefits of HIE can influence patients perception to endorse HIE. According to Simon et al. [9], the potential benefits of the electronic exchange of health information is the driving force for patients’ willingness to opt in to a HIE system. Patel et al. [12] report that the most important improvements related to use of HIE are: better communication between doctors involved in care, completeness and accuracy of medical records, safety, and overall quality of patient’s healthcare. HIE can potentially improve public health benefits through tracking of chronic diseases and early detection of infectious diseases that in some instances can be life-saving. Patients recognize several perceived benefits associated with HIE technology such as convenience, expedited care due to information sharing, high quality care, and reduced healthcare bill [22]. Therefore, patients who experience more convenient (due to reduced coping and carrying of clinical information) and expedited care delivery, eagerly support government initiatives for wide adoption and implementation of the HIE technology. Promptly available patient health information reduces delays in the process of delivering healthcare services and speeds up the physician’s decision about the best treatment and care planning. According to Park et al. [10], it is found that patients like to recommend healthcare providers who use an HIE system to friends and family members due to the convenient and expedited care process.

Patients are expected to accept having their health information shared through HIE due to perceived benefits in spite of perceived concerns about information security [13]. It declares that although patients perceive concern about information safety and security of information exchange procedures, they are willing to endorse HIE. Potential benefits of HIE such as improved quality and safety of health care can persuade them to provide consent for endorsement. Our findings also indicate that for a group of consumers, HIE is the safest method of information exchange among healthcare professionals. Consistent with O’Donnell et al. [11], patients believe that HIE can improve privacy and security of their medical records. Thus, individuals who believe that HIE will improve the privacy and security of their medical records become more likely to have their health information shared through HIE. Our findings also mention consumers whose physicians use EHR are more likely to believe that EHR and HIE will improve the quality of health care delivery and they become more willing to support HIE efforts. This perception highlights that patients might develop positive views on health IT after experience with a physician using an EHR [23]. Wang et al. [24] have stated that patients show higher attitudinal support for physician HIE compared to the physicians and medical staff who don’t use HIE due to improved communication and better access to a comprehensive understanding of their health condition. Consequently, this review shows the overall consumers’ attitudes towards providers’ use of HIE is positive. We have highlighted that perceived benefits of HIE can lead to higher patients’ participation in their care process and make them more willingness to accept and endorse use of HIE by healthcare providers.

### Perceived concerns

Our result shows that there are some concerns related to HIE. The concern about inconvenient and delayed care delivery caused by the HIE technology due to system break down, security and privacy breaches, and the concern about complex process of dealing with the system are found to be the most important perceived concerns regarding the HIE from patients’ views [10]. One of the most cited barriers to patients’ support for HIE is concerns regarding the security of transmitting personal health information over the internet. A number of articles studying consumers who perceive that technology has a negative impact on both privacy and security [14]. Consumers may be willing to support HIE but they also value elements of transparency. According to Wright et al. [25], many patients report that HIE may worsen privacy and security. Being supportive of all physicians who use HIE in their practice is different from being interested in HIE used by trusted providers. Consumers are not likely to support all physicians using HIE but they are interested in supporting HIE usage by trusted physicians due to improved care and cost savings. This difference can be justified by consumers’ concerns about privacy and security of their health information [26]. Consumers are concerned about multiple parties and organizations accessing and viewing their health information. They are also worried about their limited control over physicians who use HIE [27]. Patients who are concerned about HIE are more likely to limit disclosure of health information to providers due to privacy and security issues.

If patients’ privacy requirements are not met, many negative consequences will result [28, 29]. For instance, reduced trust in the patient-doctor relationship, increased privacy, security and confidentiality concerns, storing incomplete or inaccurate patient information in shared records, and finally wasteful investment in providing integrated health information systems [3]. Literature points out that designing a sustainable HIE system is very challenging [30] and developing it depends on public support in terms of trust in privacy and security guidelines. Such concerns make patients hide their information from doctors if they know that physicians will share it electronically.

Security of medical records is described by having safeguards in place to protect medical information [14]. There are five main reasons to explain why consumers are concerned about the security of HIE. The main factors are: misuse of health information for fraud and identity theft, posting of sensitive personal data on the internet, receiving unsolicited advertising and junk mail due to data breach, discrimination (i.e.: job recruitment), and the potential loss of personal health information. Privacy concern regarding HIE is referred to as the anxiety that unauthorized entities may access or view personal health information without patients’ permission or consent [6]. We need to answer why consumers are concerned about the privacy regarding HIE. Consumers argue that if their personal health information is used by an unauthorized entity, it is very likely that identity fraud or discrimination (denied credit or employment) will occur. When patients’ information is shared, the issue of who can access and control the data remains a significant concern [31]. Therefore, consumers need to know how exchange process takes place among providers and who will view their personal data. Privacy concerns has increased due to the growth of HIE where patient records will be available across a wider range of healthcare settings [32, 33]. Patients confer that one of the possible risks associated with HIE is that their personal health information may be accidently linked to the wrong person or released to the wrong physician due to human or technical errors.

Reports of lost data and security breach evidence may make some people become not willing to have their personal records shared electronically [34]. Privacy and security concerns still persist among patients although encryption and authorization have been adopted in HIE [24]. To remove perceived concerns, privacy and security issues need to be explicitly discussed in the HIE process to address how patient data is exchanged [35]. The following methods are important for patient regarding the security and privacy of HIE: 1) safeguards against unauthorized viewing, 2) reviewing who have viewed their medical records, 3) selecting which parts of their medical records can be shared, and 4) opting out of information being shared electronically [12]. Merely focusing on Health Insurance Portability and Accountability (HIPAA) cannot remove privacy concerns. Responsiveness to the public’s concerns regarding their health information may improve support of data networks [14]. There is a big need to present clear information about confidentiality, privacy, security measures, individual benefits/risks, and individual control choice [36, 37]. To design effective patient-centered care models, individual needs for privacy, security and trust in healthcare providers should be critically addressed. Otherwise, people may not be willing to share their medical records with healthcare providers.

### Patient characteristics

Several studies have suggested that patient related factors such as sociodemographic characteristics, health and treatment-related variables, past experience and exposure to computer and health technology are positively associated with acceptance of HIT [38]. There is a conflicting literature about the role of gender on the use of internet for health related tasks. According to a cross-sectional telephone survey, women are less likely to support physicians who participate in HIE and also women are less likely to use personal HIE [11]. On the other hand, Park et al. [10] show that male patients are more likely to endorse HIE technology than females. Although literature supports that women are more likely to report higher computer anxiety than men, previous studies show that there is no relationship between gender and patient acceptance of Consumer Health Information Technologies (CHITs) [38]. Thus, there is no consistent pattern recognized on the relationship between gender and support for HIE.

According to Caine and Hanania [32], age is the only demographic variable that affects sharing preferences. They have found that those under 46 years would share the majority of healthcare information with home healthcare providers than those over 46 years. Ancker et al. [10] also report that adults under the age of 40 years are more likely to believe that EHR improves healthcare quality. They have also showed that only adults under the age of 40 years are more likely to perceive that EHR enhances privacy. The concerns about the security of HIE become more significant among consumers between the age of 40 and 64 years old [6]. Therefore, younger consumers seem to be more likely to believe that EHR and HIE can improve both healthcare quality and privacy of medical data and are more likely to support the exchange of their health information.

Income has also appeared as a factor affecting consumers’ perceptions. Ancker et al. [7] find that people with high income (more than $100,000) are more likely to support HIE projects. O’ Donnell et al. [11] have also stated that individuals who earn $100,000 per year are more willing to visit physicians who participate in HIE. As a result, patients with relatively high income are more likely to support sharing of their health information.

Education and race are both significant predictors of willingness to share personal health information with a primary care provider. Ethnic and racial minorities have the greatest concerns about sharing personal information via HIE [6, 39]. Campos-Castillo and Anthony [40] have stated that US immigrants are more likely to withhold information from their providers mostly due to language or cultural barriers. Therefore, patients’ immigrant status may make them rely on non-disclosure to protect against perceived privacy and security risks. Consumers with a high school education or less are less likely to believe that HIE can improve healthcare quality [23]. Literature indicates that higher education leads to increased acceptance of CHITs [38]. Hence, educated care consumers are more likely to recognize the potential benefits of HIE.

Different dimensions of prior experience (i.e., computer use, having access to the internet, and knowledge about health technology) appear to be positively associated with increased HIT acceptance [38, 41]. Literature has mentioned that consumers without a doctor using an EHR are less likely to support HIE [23]. O’ Donnell et al. [11] have indicated that individuals interested in endorsing HIE use the internet frequently. People who rarely use computer due to little computer literacy and patients who are less exposed to IT, are more concerned about health information privacy and security [13]. Park et al. [10] mention that patients with the least experience with technology and information exchange are more concerned about the process of electronic exchange of health information through HIE technology. Thus, experience of computer and the internet use is significantly associated with patient perceptions of HIE.

Patel et al. [12] report that patients who are being treated for chronic disease or medical condition are significantly more likely to support physicians who use HIE. The repeated physician-seeking behaviors (especially for patients with vulnerable health conditions who need their medical information to be shared with other physicians in different locations) makes the patients exhibit higher support for HIE. Patients with chronic disease become more interested in supporting HIE if they believe that the delayed and ineffective care planning is because the physicians treating them currently do not communicate with each other. Patients with chronic illness are also very concerned about having their health information shared via HIE [39]. The vulnerable health status of patients make them seek more privacy and confidentiality protection through informed consent [3]. For instance, being identified as HIV-infected patients may make them reluctant to share their personal information [42]. Moreover, people who care for patients with chronic conditions are more likely to support physicians using HIE. O’Donnell et al. [11] indicate that individuals caring for others with chronic medical condition are more likely to support physicians using HIE.

What are the characteristics of consumers who express greater concerns and are less likely to endorse and participate in HIE? We have found consistent effect (except for gender) regarding the relationship between patient characteristics and support for HIE. Our findings show that the patient characteristics can be considered as an important factor which affect and predict public support for HIE. Thus, HIE policy makers need to consider demographic and social factors of consumers to promote widespread participation.

### Level of patient participation in HIE

Communication in health care is both demanding and multifaceted. The traditional one-way information transfer considers the healthcare provider as the expert communicator and the patient as a passive receiver of information. Patient-centered care operates based on patients’ preferences to improve patient safety and increase patient satisfaction and participation [43]. A mutual exchange of information ensures that both patients and healthcare professionals contribute to partnership [44]. If physicians do not trust the data exchange or find it not valuable, patients will not support it [19]. Greater participation from the patients in HIE initiatives can lead to higher degree of trust among all types of demographic groups. Patient acceptance, participation and trust play a critical role in patient-centered healthcare model [45]. Patients need to be more engaged in the process of data exchange in order to trust the technology and the healthcare system. Thus, patients are more likely to accept greater responsibility to manage their healthcare. They are also willing to allow their family and physicians to gain access to their health information. However, they like to block access among unaffiliated physicians who are not directly involved in their care, friends, employers, and payers [6]. Patients desire to have a list of disclosures made by treating physicians such as care planning, treatment and payment. They want to know what type of data is disclosed, who discloses it, who receives it and what the purpose of exchange is.

Education about HIE system security, authorization process for using health information, and patient rights in case of data breach and misuse of personal information can build trust in the use of HIE [6]. Physicians acknowledge that it is important to increase consumer awareness and participation in HIE to reap the potential benefits of healthcare quality [46]. However, to what extent patients can have access to their health information is another issue. According to Wiljer [47], only some relevant medical information should be presented to patients via EHR supplemented by educational materials to support the information. Literature indicates that physicians have concerns about patients viewing new clinical data before they are explained to them especially, especially if the result is abnormal and contains negative health implications [48]. Preventing patients from viewing new records can reduce patient distress caused by accessing negative clinical results [47] and help patients better comprehend the information. Some clinical information is recorded for clinicians use and such clinical documents are written in language that is not comprehensible to most patients. There are some laws that prohibit certain test results from being communicated electronically. Also, another issue is should patients be able to monitor and add to their own personal records? If limited access to patients is the appropriate solution, policies should be created to address why certain information should be excluded from patient access.

Patients can be involved in their healthcare through HIE participation [11]. Based on Murphy et al. [49], patients’ participation in care is important both for patient satisfaction and safety. Well-informed patients are more likely to follow treatment recommendations which is vital for the outcome and safety [50] . O’Donnell et al. [11] show that consumers want to be entitled to check their medical records, communicate with treating physicians and do online administrative tasks such as scheduling appointments as well as requesting referrals and refills. In patient-centered care, having patients’ access to their EHR acknowledges patients as true partners and considers possibilities for joint planning and decision making. Patients’ engagement can improve the quality of patient-provider communication which in turn will strengthen patient trust in providers [51, 52]. Patients need to realize the importance of their role in allowing their data to be shared in desirable ways that clinicians find valuable. The current HIE initiatives have been far reaching enough to engage patients in their initiatives [19]. Patient-centered model is very new and consumers’ interest in HIE is still low [53]. Although a number of recent studies focused on patient-centered model and giving more responsibility to patients, the type or model of HIE (whether a physician can send data to another physician, a physician sending data to a patient who can share it with other physicians, or a physician accessing data from other institutions) cannot affect consumers’ opinions [7]. Policy makers need to define a new consumer right and privacy protection standards according to characteristics, requirements and features of each HIE model and not according to the traditional healthcare approaches. To increase patient engagement for development and adoption of HIE, healthcare providers should change their traditional approach and treat patients as consumers who play an important role in healthcare delivery.

### Types of health information to be exchanged

Categories of health information specify that patients are less likely to share their personal and sensitive information [3]. Patients have at least some sensitive information over which they like to have particular control [54]. National Committee on Vital and Health Statistics (NCVHS) considers five categories of information as sensitive: records relating to domestic violence, genetic information, mental health information, sexual health diseases, and substance abuse [32]. The more sensitive the health information (such as HIV related records), the less likely people will become to share them [42]. Patients desire their identity such as name, address, phone number, and other unique numbers (such as social security number) to be removed from their medical information which exchanged and used within a non-clinical setting for non-medical care purposes.

Level of anonymity is found to be important because patients are more willing to share anonymous and unidentified information. According to Caine and Hanania [32], all patients reported that they would not prefer to share their entire medical records with all potential recipients under all circumstances because most patients’ records contain sensitive health information. Patients are more likely to share a large percentage of both highly sensitive and less sensitive information with their mental health provider and prefer to share very few information with researchers [32]. According to Whiddett et al. [3], patients seem to be likely to share their non-identified health information with people other than health professionals. De-identified personal health information can be shared with third parties for purposes such as research and business intelligence [55].

A survey in New Zealand indicates that patients are more willing to accept their information being shared only among healthcare professionals when its nature is less personal [3]. Patients agree that HIE should facilitate dissemination of health information such as medication, chief complaints, lab results and diagnostic imaging which are not personal or sensitive [46, 56]. As stated by Campos-Castillo & Anthony [40], consumers are more likely to endorse exchange of de-identified information for research to remove the concern of potential breach of privacy. Patients consider some information sensitive and therefore, they may not want to share it in the same way the less sensitive information is shared [1]. This will also result in lack of complete information which is identified as a barrier to HIE usage [46]. This is crucial that patients can maintain privacy control over what they consider to be sensitive [32]. Better policies should be devised to make the current information sharing practices transparent for patients to indicate how and why the sensitive or less sensitive information will be shared. Expanding the number of institutions, providers and patients participating in HIE can improve the completeness of information and physicians’ overall satisfaction with HIE accordingly.

### Types of recipients (With whom patient data should be shared)

Classes of recipients indicate that patients are more likely to have their health information distributed to health professionals and they are less willing that their medical information shared with other stakeholders such as administrators, government departments or researchers [3]. Patients usually want to have full access to all categories of their own information and they are less likely to see their information shared with private health insurers and government agencies. Patients are unwilling to have their information released for purposes other than clinical care [3]. Both qualitative and quantitative studies support a significant relationship between the use of HIE systems for clinical and care purposes [7]. However, patients may be unwilling to allow their sensitive information shared with physicians even when it is vital to their treatment if they are not sure about confidentiality of their information [3]. Online electronic exchange of health information with health insurance plans and companies for non-clinical purposes such as research, or marketing receives less support [57]. Whiddett et al. [3] indicate that patients are more likely to have more restrictions placed on the exchange practice among healthcare providers especially for purposes unrelated to clinical care provision. They also mention that patients may also become willing to share their information with health administrators and researchers if they are consulted first.

As stated by Dimitropoulos et al. [6], consumers are in need of greater transparency and control about who will view and access their information and to whom it will be shared. HIE mechanism offered by healthcare providers is more trustworthy to patients than PHRs offered by insurers or large companies such as Microsoft and Google [7]. Type of organization conducting the medical research affects the likelihood of obtaining consent to share information in a research network. Individuals are most likely to consent if asked by a hospital and least likely if asked by an insurance company. Trust in healthcare providers is a significant factor affecting consumers whether to participate in data sharing [58]. A study shows that in case of patients living with HIV, there is a strong correlation between trust and willingness to share personal health information with primary treating clinicians [42]. However, this relationship becomes insignificant in case of sharing medical information with non-clinical staff, community providers and public health [14].

According to Caine and Hanania [32], the majority of patients are more likely to share all their health information with primary physicians and emergency medical providers but not with non-treating physicians. Patients indicate that they like to give temporary and limited access to certain recipients (such as government agencies or various physicians) based on need [32]. Many patients do not want to share private information if it is not completely required for their medical care. As discussed by Teixeira et al. [42], the majority of individuals living with HIV/AIDS agree that their personal health information shared only with clinicians involved in their care including their primary care providers and disagree to share their clinical information with non-clinical staff. Purpose of data sharing is positively associated with willingness to share. Grande et al. [59] report that marketing purposes have achieved lowest patient willingness to share their health information. Clear and understandable privacy policies should be provided to describe who may access patients’ personal information under what circumstances.

### Patients’ preferences regarding consent

Literature shows that consent and permission of viewing medical records are important to patients [60]. For instance, Patel et al. [12] indicate that the majority of consumers allow the doctors and providers who are involved in their treatment to view their records electronically via HIE only with their permission except in medical emergency conditions. The right of informed choice and consent is violated when many healthcare providers assume that patients have implicitly given consent to distribute their health information by seeking their services [17]. In this case, healthcare providers rarely get further clarification when sharing patients’ health-related information with other providers [3]. However, the process of dealing with informed consent and consultation to access patient information should be handled carefully to avoid increasing the workload of healthcare professionals.

According to Coiera and Clarke [18], there are four main forms of consent: 1) General consent: it is assumed that patients have given an inclusive consent for their information to be used and shared in all cases and instances; 2) General consent with specific denial: patients place some limitations and restrictions on using and distributing their health information. It applies many restrictions in case of sharing patient information for the purpose other than provision of care (such as research). Therefore, by using this form of consent, healthcare professionals can use and exchange patient information for purposes of care delivery within the clinical setting. This form of consent also allows patients to limit access to some sensitive medical information; 3) General denial with specific consent: patients do not generally allow providers to access their information except in some specific circumstances such as for care purposes. In general, healthcare providers are banned from using and exchanging the patient health information unless a permission granted by them for care provision purposes. In this case, patients are fully aware of how their information will be used. It requires obtaining a great deal of consents from patients on an ongoing basis; 4) General denial: healthcare providers require consent from patients to use or share their information on each occasion. It enforces a very severe access control protocol to ensure privacy requirements.

The consent process can also be described in two forms: opt-in and opt-out systems. An opt-in system gives patients the ability to participate in HIE by providing explicit consent so that they can allow or avoid HIE. In opt-in policy, prior to uploading patient’s data from a physician’s office EHR to the HIE community database, a signed patient consent form is required in all cases [8]. A specific type of Opt-in form is Opt-in with break the glass which allows access to data without agreement only in an emergency [14]. An opt-out system assumes that there is no need to obtain explicit consent from patients for the purpose of information exchange. In opt-out model clinical data can be shared over the network unless a patient formally requested otherwise [19]. According to Simon et al. [9], patients are more willing to participate in opt-in rather than opt-out HIE systems. There are two drivers for choosing an opt-in approach. The first one is due to strict privacy laws that lead to conservatism in the face of uncertainty. The second driver is the feedback from consumers. Increasing concern about privacy and security of clinical data requires new technologies to allow patients to be stewards of their own medical information [19].

Paper-based consent may result in an additional work burden due to frequent human contact [7]. Therefore, e-consent is a solution to patient consent concern by which patients agree to share their medical information with other hospitals in case of clinical needs when their privacy is ensured [61]. Consent forms are written by the health care organizations seeking the data and are often termed in general or vague words that highlight potential uses of the data. Therefore, consumers may authorize access to their personal health information via a consent form or policy that they do not fully comprehend. A new mandate should describe the terms of HIE service and the privacy policies in a meaningful way not just with very general and unclear terms. Different consent forms should be obtained from patients for disclosing their personal data for health care, marketing or research purposes [55]. Most people do not read the details of consent forms and just assume that the presence of privacy terms and conditions means their data will not be shared at all [62]. Many consent forms are not very informative and written in a way to obtain patient consent for all potential uses of data [63]. People are usually asked for a consent when they are in hospitals waiting for a vital treatment or when they apply for insurance. Under these circumstances, it is not very likely that people say no to authorization if they perceive that the treatment or insurance coverage may be cancelled. They, then, authorize the disclosure of information for all potential uses and purposes and third parties mine their sensitive data based on the initial authorization [55]. This fact highlights deficiencies in current consent guidelines and serves as an evidence for a new policy approach to educate patients on consent forms and features.

## Study contributions

### Theoretical contributions: a guiding framework

**Fig. 4:**
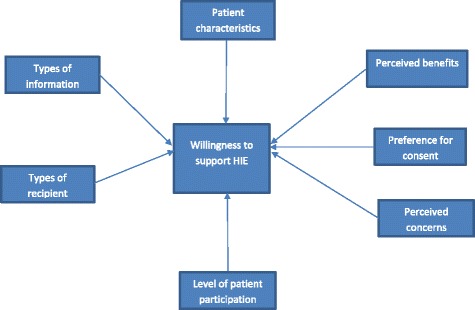
Guiding framework

It is clear from our review that consumer support has been viewed as a vital factor in the successful implementation of HIE. Therefore, scholars have been most interested in identifying drivers and barriers which affect consumer willingness to endorse HIE. Based on reviewing a body of HIE literature, this study has described the main variables of interest. Using our literature review as the basis as well as mentioned classification and argumentation, we have summarized all of our observations into one framework. To reflect theoretical implications from the synthesis of this review, a guiding framework has been developed. This guiding framework (Fig. 4) helps researchers better understand the factors affecting consumer willingness to support HIE.

The healthcare system in US is highly fragmented [64]. As a result, healthcare costs are higher and patient health outcomes are negatively affected [65]. Due to the lack of continuity of care, physicians have limited information to make clinical decisions and it may endanger patient safety [66]. Limited information at the point of care causes healthcare service duplication such as laboratory tests and duplication of therapy. Furthermore, administrative cost becomes higher due to the need to search for the missing information and it causes delays in providing care which in turn leads to lower efficiency of healthcare providers. The concept of HIE claims that if providers get access to additional patient clinical information, their decision making process and ultimately patient outcomes will be improved [46]. The process of obtaining health records via HIE is faster than obtaining them from other providers or directly from the patients. However, HIE can reduce the providers’ work efficiency if the required health information is not available. Improvement in data availability at the point of care is perceived as a significant impact on patient safety and quality of healthcare delivery [46].

HIE is successful only when patients are willing to share their medical data [19]. Consumers may conceptually support the development of HIT and infrastructures which enable HIE but it is not guaranteed that they will exchange their personal health information [12]. Literature indicates that there are a few studies on consumers’ support and preferences regarding HIE and little is known about how individuals are interested in exchanging their health records [67]. This study contributes to knowledge by providing a better picture of why consumers will support or reject HIE initiatives. Our proposed guiding framework (which is originated from the literature) addressed the research question by describing the seven main factors which are driven by literature. According to our literature review, we have discussed that patients consider the process of electronic collection, storage and dissemination of their health information beneficial due to provision of better care [9]. Patients are willing to support HIE but they also value elements of transparency, privacy and security issues such as individual control, who can access, and the purpose for use of data. Patients start developing favorable opinions about HIE when they realize that, by using HIE, physicians can help improve the quality, safety, efficiency, and affordability of care. In contrast, if they experience data breaches or notice that their physicians are distracted by technology they will develop negative opinions toward HIE [23]. Thus, educational efforts are required to furnish the public with accurate information on benefits and concerns associated with HIE [10]. As a result, if patients are aware of potential benefits and concerns related to data sharing, they are more likely to support HIE initiatives. Patient engagement is vital to HIE efforts in determining how much information is shared and how it is shared [19]. HIE business model will require a long-term connection between patient engagement and clinicians. The sustainability of HIE hinges upon how much patient information is stored in the system regarding breadth (number of patient records included) and depth (amount of information in each patient record) [19]. Therefore, in line with the literature, we argue that patient participation will result in patient willingness to support HIE.

Patient characteristics such as prior experience using the internet for managing healthcare, college education, income and age can affect their perception of how HIE can influence quality and safety of healthcare. Personal privacy right is an important principle in many cultures and the use of communication technologies can be threatening to people’s privacy [3]. Patients with chronic illness and patients from racial and ethnic minorities are more concerned about privacy and security of their digital health records [55, 68]. Some studies indicate that males are more likely to support HIE than females but the mechanism has not been articulated [69]. Or and Karsh [38] mention that females report more computer anxiety and concern about data breach. Therefore, according to our review, we argue that consumers’ demographic characteristics can impact level of support for HIE.

Finally, HIE support is affected by factors such as identity of recipient, type of information and consent provision. Patients are concerned about their highly sensitive medical information that may be shared with other recipients. Patients report that they are not well informed of how their information is shared whereas they prefer to be consulted about the exchange of their information [3]. Patients prefer to share information if used for their healthcare benefit, otherwise they like to keep it private [32]. Patients should be aware of the current information sharing practices between healthcare providers to convert an implied consent to an informed one [70]. Corresponding to the literature, we also argue that establishing clear consent procedures and providing transparency related to HIE process (including type of medical records and recipients) will affect consumers’ decision to support HIE.

### Practical contributions: call for new strategies

The lack of clarity in federal and state law related to the area of sharing health information affects patients’ attitude toward HIE. Federal and state laws in design and policy parameters are relatively general and in some cases difficult to interpret and apply [19]. It is important to study patient willingness to share their clinical information and identify factors associated with willingness to endorse HIE. Factors affecting consumer attitudes towards HIE can help policy makers and HIE vendors shape efforts to improve consumer support for the implementation of HIE.

Literature has reported generally favorable public opinions toward HIE accompanied by strong privacy and security concerns [23]. National efforts and policies to promote health IT have not been able to resolve privacy issues yet. State privacy laws are fragmentary and inconsistent and cannot provide the appropriate privacy assurance for both developers and consumers [55]. The design of HIE systems should ensure appropriate access control policies to meet the preferences of patients. Having a right balance between patients’ personal privacy against the potential use of their health-related information for the purpose of care improvement is required in the health care sector. Policymakers need to establish more transparent privacy and security policies to address concerns expressed by various demographic groups. However, current health policies mostly support healthcare organizations that provide care for underserved populations.

Having more trust in healthcare providers and the technology vendor will result in increased acceptance of technology. A solution to earning the consumer trust is establishing consumer councils to voice and address their pressing concerns such as privacy and consent issues. Some significant policy and design considerations especially, in two areas of privacy and security (consent management and authorization) and data sharing (which parts of the record to share) need to be established before the HIE networks are deployed [19]. Policy makers can streamline the consent process through electronic communication such as creating an e-consent process. Local HIE initiatives can establish committees consisting of physicians, hospital leaders, other healthcare professionals and consumer representatives to reconcile privacy and security issues. Designers should provide interfaces for patients to express privacy and sharing preferences. Use of personal health information for marketing purposes without individual authorization is a key privacy concern. Tighter restrictions on marketing are needed to ensure that the personal information will not be used without their authorization to market goods and services to them [55]. To build the public trust in HIE, core privacy principles should be implemented, design characteristics for a trusted network should be adopted, and accountability and oversight mechanism should be established. New regulatory standards need to prevent certain uses of patient genetic information for some nonmedical purposes such as employment, credit, or insurance even with consent. Additional policies are required to articulate how and when consent is obtained and how the information will be used. New policies should give more weight to individual’s right to limit access to some sensitive data.

Policy makers need to discuss the positive side (improvement in quality of care) and the negative side (privacy and security concerns) of HIE to clarify the technology’s potential. Privacy and security policy should concentrate on protecting health information via rules, requirements, and system standards. Policy makers should address evidence of data breaches, unauthorized access and questionable use of data and how these instances can be prevented in order to protect patient confidentiality [40]. They also need to raise the awareness of consumers on HIE takes place among providers. Different strategies are required for educating consumers with differing demographic characteristics and health status [9]. Additional policies may be required to enlighten patients about responsibilities of different stakeholders involved in different aspects of electronic exchange. Policy makers need to communicate the goals and risks of HIE and encourage consumer participation. If patients are aware of the benefits of exchange, they become more willing to participate and allow their medical data to be shared. To increase consumer participation in HIE, consent forms, educational brochures and frequently asked questions (FAQ) should be professionally drafted [19]. Professional marketing approaches can turn permission to demand. To do so, marketing approaches should find and address what consumers are most frustrated with, what concerns them the most, and if the consent forms can convey the benefits and risks of their decision. Marketing approaches can also make the most important HIE benefits such as convenience, safety and quality of care right up front to make them bold and memorable. In order to build the trust of consumers and address data security concerns, HIE security safeguards can be compared to security provisions that banking intuitions have adopted.

## Limitations and future studies

Like other studies, this article has limitations. First, caution should to be exercised in interpreting the classification and results due to the procedure of selecting included studies and classification method. The inclusion criteria and keywords chosen for conducting this review also limited the inclusion of all potentially relevant research. Other selection criteria and broader classification scheme may have led to a more comprehensive classification. Furthermore, only five main electronic research databases were searched for reporting articles. Thus, using other databases may have included additional studies and broader reflection of the current literature. Second, gray literature such as secondary analyses, reports, and dissertations were not reported in this study and only peer-reviewed publications were included. Therefore, there is a potential for introducing possible publication bias. Third, we summarized our findings and developed a framework based on the key factors extracted from the literature. This framework is a theoretical guideline constructed according to the studies reported in this review and may have limited generalizability as compared to a conceptual model which precedes a search strategy. Moreover, the framework can be tested by future studies on the basis of empirical data using statistical methods. Finally, it should be noted that this review was aimed at highlighting a summary of the existing literature on sharing health information from patients’ views and their willingness to support HIE. Despite the mentioned limitations, the findings of this article can help future studies and policy makers better understand consumers’ preferences regarding HIE mechanism and encourage them to support HIE usage among physicians and providers.

## Conclusion

Assessing the preferences of patients in the process of exchanging their medical records can predict their acceptance and support for HIE. The widespread adoption of HIE depends on engaging patients and winning their trust. A large proportion of patients prefer to get access to their health information and also want to share it with their care providers to improve the quality of their care. However, they are concerned about identity theft or fraud and misuse of their medical records for other purposes such as marketing and research. Although privacy preferences, sensitivity perceptions and perceived potential benefits vary from person to person, patients’ perceptions of the pros and cons of HIE can highly influence their level of engagement in data exchange and endorsing HIE. Therefore, additional effort should be made towards educating all types of demographic groups to clearly articulate the potential advantages and risks associated with HIE. New policy approaches are required to highlight the benefits of HIE in treatment and care coordination and alleviate possible concerns such as privacy and security issues. Policy makers should devise new strategies to ensure patients’ rights to informed consent to better address the real and perceived privacy and security risks of HIE. By doing so, patients are more likely to endorse HIE when they believe that the potential benefits far outweigh the possible risks.

## Additional files

Additional file 1: Full search strategy: this file contains details of full search strategy in the five databases. (DOCX 13 kb)

Additional file 2: Quality scoring of included studies: this file contains details of the scores corresponding to the four quality assessment factors for each of the 36 studies included in the analysis. (DOCX 16 kb)

## Abbreviations

CDSS: Clinical decision support system
CHITs: Consumer health information technologies
CPOE: Computerized physician order entry
EHRs: Electronic health records
HIE: Health information exchange
HIPAA: Health insurance portability and accountability
HIT: Health information technology
HITECH: The health information technology for economic and clinical health act
NCVHS: National committee on vital and health statistics
PHRs: Personal health records
