# Supplementary material for: Patients’ support for health information exchange: a literature review and classification of key factors
Source: BMC Med Inform Decis Mak. 2017 Apr 4;17:33. doi: 10.1186/s12911-017-0436-2 (PMC5379518; doi:10.1186/s12911-017-0436-2)
Supplement: Supplementary file 1 — Full search strategy: this file contains details of full search strategy in the five databases. (DOCX 13 kb) [file 12911_2017_436_MOESM1_ESM.docx]

Full search strategy:

Terms: “Health Information Exchange”, “HIE”, “Patients”, “Consumers”, “Participation”, “Involvement”, “Role”, “Attitude”, “Experience”, “View”, “Concerns”, and “Benefits”

1. " Health Information Exchange " AND "Patients"
2. " Health Information Exchange " AND “Consumers"
3. "HIE" AND " Patients "
4. "HIE"AND " Consumers "
5. " Health Information Exchange " AND "Patients" AND “Role”
6. " Health Information Exchange " AND "Patients" AND “Participation”
7. " Health Information Exchange " AND "Patients" AND “Involvement”
8. " Health Information Exchange " AND "Patients" AND “Attitude”
9. " Health Information Exchange " AND "Patients" AND “Experience”
10. " Health Information Exchange " AND "Patients" AND “View”
11. " Health Information Exchange " AND "Patients" AND “Concerns”
12. " Health Information Exchange " AND "Patients" AND “Benefits”
13. " Health Information Exchange " AND " Consumers" AND “Role”
14. " Health Information Exchange " AND " Consumers " AND “Participation”
15. " Health Information Exchange " AND " Consumers " AND “Involvement”
16. " Health Information Exchange " AND " Consumers " AND “Attitude”
17. " Health Information Exchange " AND " Consumers " AND “Experience”
18. " Health Information Exchange " AND " Consumers " AND “View”
19. " Health Information Exchange " AND " Consumers " AND “Concerns”
20. " Health Information Exchange " AND " Consumers " AND “Benefits”
21. " HIE " AND "Patients" AND “Role”
22. " HIE " AND "Patients" AND “Participation”
23. " HIE " AND "Patients" AND “Involvement”
24. " HIE " AND "Patients" AND “Attitude”
25. " HIE " AND "Patients" AND “Experience”
26. " HIE " AND "Patients" AND “View”
27. " HIE " AND "Patients" AND “Concerns”
28. " HIE " AND "Patients" AND “Benefits”
29. " HIE " AND " Consumers" AND “Role”
30. " HIE " AND " Consumers " AND “Participation”
31. " HIE " AND " Consumers " AND “Involvement”
32. " HIE " AND " Consumers " AND “Attitude”
33. " HIE " AND " Consumers " AND “Experience”
34. " HIE " AND " Consumers " AND “View”
35. " HIE " AND " Consumers " AND “Concerns”
36. " HIE " AND " Consumers " AND “Benefits”
37. " Health Information Exchange " AND "Patients" AND “Benefits” OR “Concerns”
38. " Health Information Exchange " AND " Consumers " AND “Benefits” OR “Concerns”
39. " HIE " AND "Patients" AND “Benefits” OR “Concerns”
40. " HIE " AND " Consumers " AND “Benefits” OR “Concerns”
