# Supplementary material for: Patients’ support for health information exchange: a literature review and classification of key factors
Source: BMC Med Inform Decis Mak. 2017 Apr 4;17:33. doi: 10.1186/s12911-017-0436-2 (PMC5379518; doi:10.1186/s12911-017-0436-2)
Supplement: Supplementary file 2 — Quality scoring of included studies: this file contains details of the scores corresponding to the four quality assessment factors for each of the 36 studies included in the analysis. (DOCX 16 kb) [file 12911_2017_436_MOESM2_ESM.docx]

Quality scoring of included studies:

| # | **Reference** | **Study**  **Type ^a^** | **Sampling ^b^** | **Method**  **Detail ^c^** | **Analysis ^d^** | **Total Score** |
| --- | --- | --- | --- | --- | --- | --- |
| 1 | Agaku et al. (2014) [27] | 4 | 3 | 1 | 3 | 11 |
| 2 | Ancker et al. (2012) [7] | 4 | 3 | 1 | 2 | 10 |
| 3 | Ancker et al. (2013) [23] | 4 | 3 | 1 | 3 | 11 |
| 4 | Beard et al. (2012) [31] | 3 | 0 | 1 | 1 | 5 |
| 5 | Caine & Hanania (2013) [32] | 5 | 3 | 1 | 3 | 12 |
| 6 | Campos-Castillo & Anthony (2015) [40] | 4 | 3 | 1 | 3 | 11 |
| 7 | Chhanabhai & Holt (2007) [29] | 4 | 3 | 1 | 3 | 11 |
| 8 | Dhopeshwarkar et al. (2012) [70] | 4 | 3 | 1 | 3 | 11 |
| 9 | Dimitropoulos & Rizk (2009) [60] | 3 | 0 | 0 | 1 | 4 |
| 10 | Dimitropoulos et al. (2011) [6] | 4 | 3 | 1 | 3 | 11 |
| 11 | Furukawa et al. (2014) [53] | 4 | 3 | 1 | 2 | 10 |
| 12 | Galpottage & Norris (2005) [61] | 3 | 0 | 0 | 1 | 4 |
| 13 | Grande et al. (2013) [59] | 4 | 3 | 1 | 3 | 11 |
| 14 | Hincapie et al. (2011) [46] | 3 | 2 | 1 | 2 | 8 |
| 15 | Kaelber & Bates (2007) [66] | 3 | 0 | 0 | 1 | 4 |
| 16 | Kim et al. (2015) [14] | 4 | 3 | 1 | 3 | 11 |
| 17 | Kullberg et al. (2015) [50] | 4 | 3 | 1 | 2 | 10 |
| 18 | Letrilliart et al. (2009) [43] | 4 | 3 | 1 | 2 | 10 |
| 19 | McGraw et al. (2009) [55] | 3 | 0 | 0 | 1 | 4 |
| 20 | O’Donnell et al. (2011) [11] | 4 | 3 | 1 | 2 | 10 |
| 21 | Or & Karsh (2009) [38] | 3 | 0 | 1 | 2 | 6 |
| 22 | Park et al. (2013) [10] | 4 | 3 | 1 | 3 | 11 |
| 23 | Patel et al. (2011) [68] | 4 | 3 | 1 | 2 | 10 |
| 24 | Patel et al. (2012) [12] | 4 | 3 | 1 | 3 | 11 |
| 25 | Shield et al. (2010) [45] | 3 | 3 | 1 | 2 | 9 |
| 26 | Simon et al. (2009) [9] | 3 | 3 | 1 | 2 | 9 |
| 27 | Tang et al. (2006) [58] | 3 | 0 | 0 | 1 | 4 |
| 28 | Teixeira et al. (2011) [42] | 4 | 3 | 1 | 3 | 11 |
| 29 | Tripathi et al. (2009) [19] | 3 | 0 | 0 | 1 | 4 |
| 30 | Unertl et al. (2012) [22] | 3 | 3 | 1 | 2 | 9 |
| 31 | Vest & Gamm (2010) [1] | 3 | 0 | 0 | 1 | 4 |
| 32 | Wang et al. (2015) [24] | 4 | 3 | 1 | 3 | 11 |
| 33 | Wen et al. (2010) [28] | 4 | 3 | 1 | 3 | 11 |
| 34 | Whiddett et al. (2006) [3] | 4 | 3 | 1 | 3 | 11 |
| 35 | Wiljer et al. (2008) [47] | 3 | 3 | 1 | 2 | 9 |
| 36 | Wright et al. (2010) [25] | 4 | 3 | 1 | 3 | 11 |
| Range | | 3-5 | 0-3 | 0-1 | 1-3 | 4-12 |
| Mean | | 3.64 | 2.22 | 0.81 | 2.22 | 8.92 |

^a^ Study design scores: 3=qualitative design; 4=quantitative descriptive design; 5=mixed qualitative and quantitative descriptive; 6=quantitative experimental and quasi-experimental

^b^ Sampling (for primary study aim): 0=Not explained; 1=Convenience; 2=Purposive or Case matching/cohort; 3=Random or 100%

^c^ Method detail: 1=Methods and tools; 0=Not explained

^d^ Analysis: (highest level reported): 1=Narrative; 2=Descriptive statistics; 3=Inferential statistics.
